# Metabolic Responses of *Eisenia Fetida* to Individual Pb and Cd Contamination in Two Types of Soils

**DOI:** 10.1038/s41598-017-13503-z

**Published:** 2017-10-12

**Authors:** Ronggui Tang, Changfeng Ding, Yibing Ma, Junsong Wang, Taolin Zhang, Xingxiang Wang

**Affiliations:** 1 Key Laboratory of Soil Environment and Pollution Remediation, Institute of Soil Science, Chinese Academy of Sciences, Nanjing, 210008 People’s Republic of China; 2 Institute of Agricultural Resources and Regional Planning, Chinese Academy of Agricultural Sciences, Beijing, 100081 People’s Republic of China; 3 Center for Molecular Metabolism, School of Environmental and Biological Engineering, Nanjing University of Science and Technology, Nanjing, 210014 People’s Republic of China; 4 University of the Chinese Academy of Sciences, Beijing, 100049 People’s Republic of China

## Abstract

To characterize the potential toxicity of low Pb- and Cd-contaminated arable soils, earthworms were exposed to Pb contaminated ferrosol, cambosol or Cd contaminated ferrosol for two weeks. Polar metabolites of earthworms were detected by nuclear magnetic resonance. Data were then analyzed with principal component analysis followed by orthogonal signal correction-partial least squares-discriminant analysis and univariate analysis to determine possible mechanisms for the changes in metabolites. The survival rates, metal concentrations and bioaccumulation factor (BAF) of the earthworms were also measured and calculated as auxiliary data. The results showed that the metabolite profiles were highly similar in Pb-contaminated ferrosol and cambosol (R^2^ = 0.76, *p* < 0.0001), which can be attributed to similar response mechanisms. However, there was a more intense response in ferrosol likely due to higher Pb concentrations in earthworms. Metabolic pathways and BAFs exhibited apparent distinctions between Pb- and Cd-contaminated ferrosol, likely because they bind to different bio-ligands. The affected metabolic pathways were involved in alanine-aspartate-glutamate, purine, glutathione, valine-leucine-isoleucine biosynthesis and degradation and nicotinate and nicotinamide metabolism. Regarding the bioavailability in earthworms, Pb availability was higher for ferrosol than for cambosol. We confirmed that the potential toxicity of low Pb/Cd-contaminated soils can be characterized using earthworm metabolomics.

## Introduction

The heavy metal pollution of soils directly or indirectly influences animal and human health via food chain transference and accumulation^1,2^. In China, the overall situation of the national soil environment is not optimistic and the situation regarding Cd- and Pb-contaminated arable soils is particularly serious, with mainly moderate and low pollution according to Bulletin National Survey of Soil Pollution (http://www.zhb.gov.cn/gkml/hbb/qt/201404/t20140417_270670.htm).

Earthworms are an essential part of the soil fauna in most soil globally, and they constitute a significant proportion of soil biomass^3^. Traditionally, earthworms have been used as model organisms to gauge soil ecosystem health and predict the potential influence of xenobiotics by investigating their apparent responses to external contaminants in soil^4,5^. According to the Organization for Economic Co-operation and Development (OECD) guidelines, heavy metal accumulation in earthworms^6–8^ and the induced mortality rate, cocoon production and growth of earthworms have been widely studied^9–11^. However, these toxic parameters are difficult to connect to the toxicity of low heavy metal concentrated soils in the real world and clarify toxicity mechanisms at the molecular level.

Metabolomics, a new analytical approach, mainly focuses on small molecular weight metabolites (<1000 Da), which are the end products of the metabolic processes of a variety of biological systems^12^. The levels of metabolites of organisms under suitable conditions should be stable and kept within a certain range until environmental conditions change (e.g., contaminant exposure)^13^. Metabolomics has the particular potential for defining biomarkers that predict the severity of environmental pollutants^14^. In recent years, ^1^H nuclear magnetic resonance (NMR)-based metabolomics has gained popularity as a powerful tool for measuring organismal responses to various environmental pollutants, and has helped reveal the toxicological mechanisms of pollutants and identify broader ranges of biomarkers on the molecular level^15,16^. The toxicological effects to earthworms of a broad spectrum of toxicants have been examined by culture experiments on filter paper or artificial soils, including organics (polycyclic aromatic hydrocarbons, polychlorinated biphenyl)^17–19^, farm chemicals (dichlorodiphenyltrichloroethane, atrazine)^20,21^ and new materials (buckminsterfullerene, nano titanium dioxide)^17,22^. For trace metals, reports show that nickel causes changes in fumarate, lysine and myo-inositol in earthworms^23^, and copper can interfere with energy metabolism, decrease the levels of glucose and mannose^24^ and increase the levels of histidine^25^. However, soil types are not considered in terms of the exposure media in these studies, and the metabolic responses of earthworms to low Pb-and Cd- contaminated soils are seldom reported. In reality, it is difficult to find a series of ideal historically polluted soils for studies^26^. Long-term aged heavy metal contaminated soils (to some extent, also historically contaminated soils) can reach an equilibrium state close to that of actual contaminated soil^27,28^. In addition, the toxicity of heavy metals is related to the their bioavailability and different soil types due to their distinguishing physical and chemical characteristics such as pH^29,30^, and organic matter^31,32^ causes differences in the bioavailability of heavy metals^33,34^. Therefore, the real metabolic toxic responses of earthworms to different soil types with low heavy metal contamination (at environmentally relevant concentrations) should be explored.

Earthworm biomarkers for the biological indicator method are used to indicate the status of soil environmental pollution. Metabolic biomarker research into long-term aged low heavy-metal -contaminated soils can objectively reflect the real bioavailability and toxicity of soil heavy metals and enable comprehensive evaluation of the risk of soil pollution. With increasing concern about soil heavy metal pollution, it will be of great importance to the public, media and policy-making departments to understand the potential toxic effects of low heavy-metal-contaminated soils. Furthermore, identifying the toxicity of heavy metal contaminated soils with low concentrations or environmentally relevant levels by significantly changing biomarkers is critical to provide an early warning for soil environmental protection. In this work, earthworm exposure experiments in long-term-aged Pb/Cd-contaminated ferrosol and Pb-contaminated cambosol were conducted and ^1^H NMR-based metabolomics approach was employed to characterize the initial toxic responses.

Our objectives were (1) to find similarities and differences in the metabolic pathways of earthworms exposed to Pb-contaminated ferrosol and cambosol and characterize the metabolic responses qualitatively (different soil types); (2) to characterize the metabolic responses of earthworms exposed to both Pb- and Cd-contaminated ferrosol (different heavy metals); and (3) to study the bioavailability of Pb in ferrosol and cambosol to earthworms using a metabolomics approach.

## Results

### Earthworm metal concentrations and BAF

**Figure 1:**
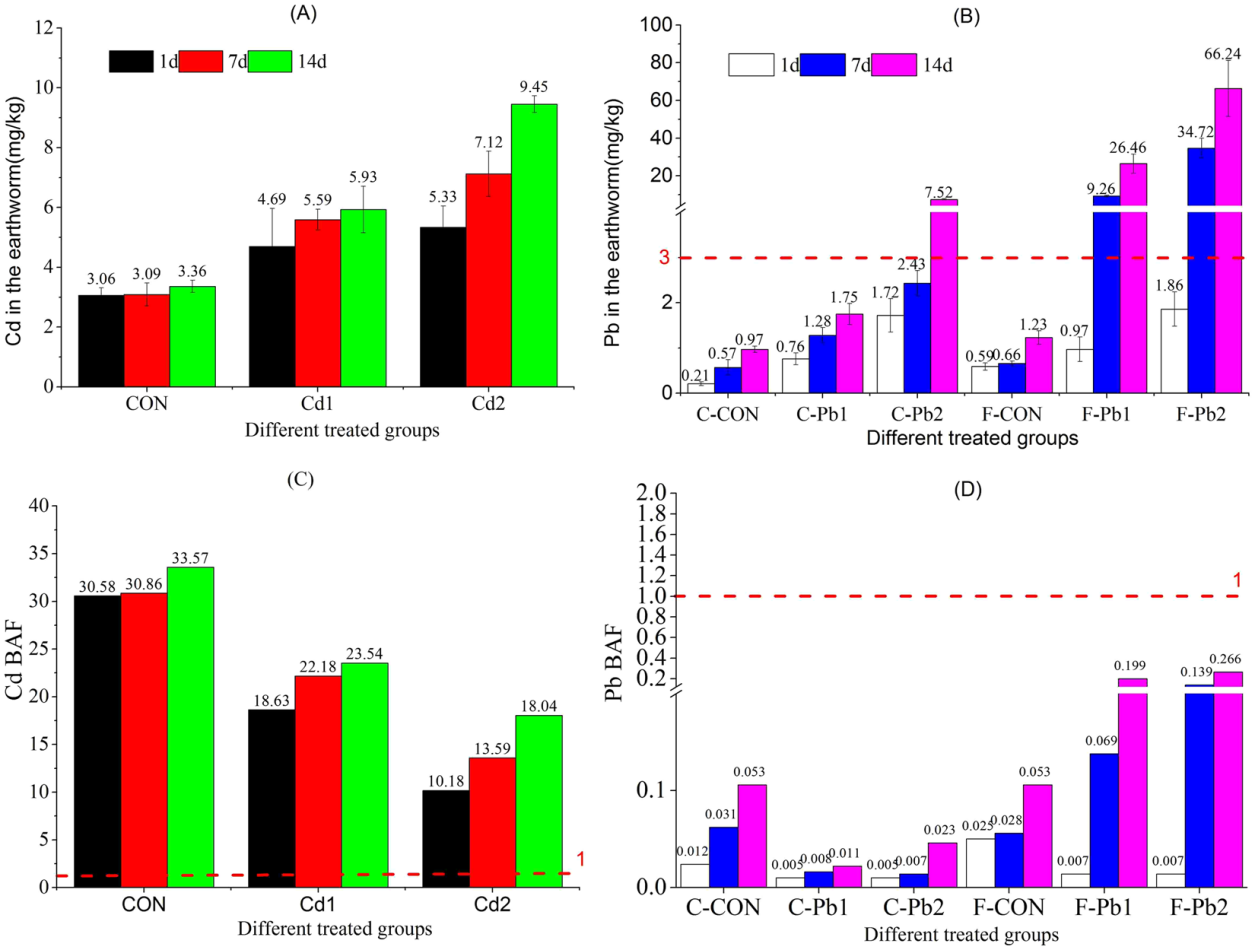
Cd and Pb concentrations of earthworms in different treated groups with changing time and bioaccumulation factor (BAF) for Cd- and Pb- treated groups during the exposure process. F-CON, F-Cd1and F-Cd2 stand for Cd treated groups (CON, Dose 1 and Dose 2) in ferrosol, and F-CON, F-Pb1, F-Pb2 and C-CON, C-Pb1, C-Pb2 represent Pb treated groups (CON, Dose 1 and Dose 2) in ferrosol and cambosol.

The survival rates of earthworms after exposure were 97–100% in each group. Individual earthworms died in some beakers independently of the exposure scenario. The earthworm metal (Pb and Cd) concentrations are presented in Fig. 1. In control groups, heavy metal concentration in the earthworms were not strongly affected by exposure time, whereas exposure time affected the internal concentrations of earthworms in Cd- and Pb-contaminated soils. With longer exposure times, the Cd concentrations in earthworms in the control group were maintained at approximately 3 mg/kg, whereas the Cd concentrations of the Cd1 and Cd2 groups were 4.69, 5.59, 5.93 and 5.33, 7.12, 9.45 mg/kg, respectively, on the 1^st^, 7^th^ and 14^th^ day (Fig. 1A). Accordingly, the Cd concentrations of the earthworm increased with increasing Cd concentration in the soil. Similarly, the Pb concentrations in the earthworms in the Pb1 and Pb2 groups on cambosol and ferrosol significantly increased with longer exposure and were 0.76, 1.23, 1.75(Pb1-C), 1.72, 2.43, 7.52 (Pb2-C) and 0.97, 9.26, 26.46 (Pb1-F) and 1.86, 34.72, 66.24 (Pb2-F), respectively on the 1^st^, 7^th^ and 14^th^ day (Fig. 1B). Furthermore, the Pb concentrations in the earthworms in ferrosol were much higher than that in the earthworms in cambosol. The water content of all earthworms was 81.8–84.5% (n = 70). During the 14 days of exposure, the BAF values of Cd-exposed groups in ferrosol were all above 10 (10.13–33.57) (Fig. 1C), whereas the BAF values of Pb exposed groups in ferrosol and cambosol were all less than 0.3 (0.005–0.266), and the Pb BAF in ferrosol was much greater than that in cambosol (Fig. 1D). In other words, Cd BAF was at least 30-fold higher than Pb BAF in this study.

### ^1^H NMR metabolic profile analysis of earthworms

**Figure 2:**
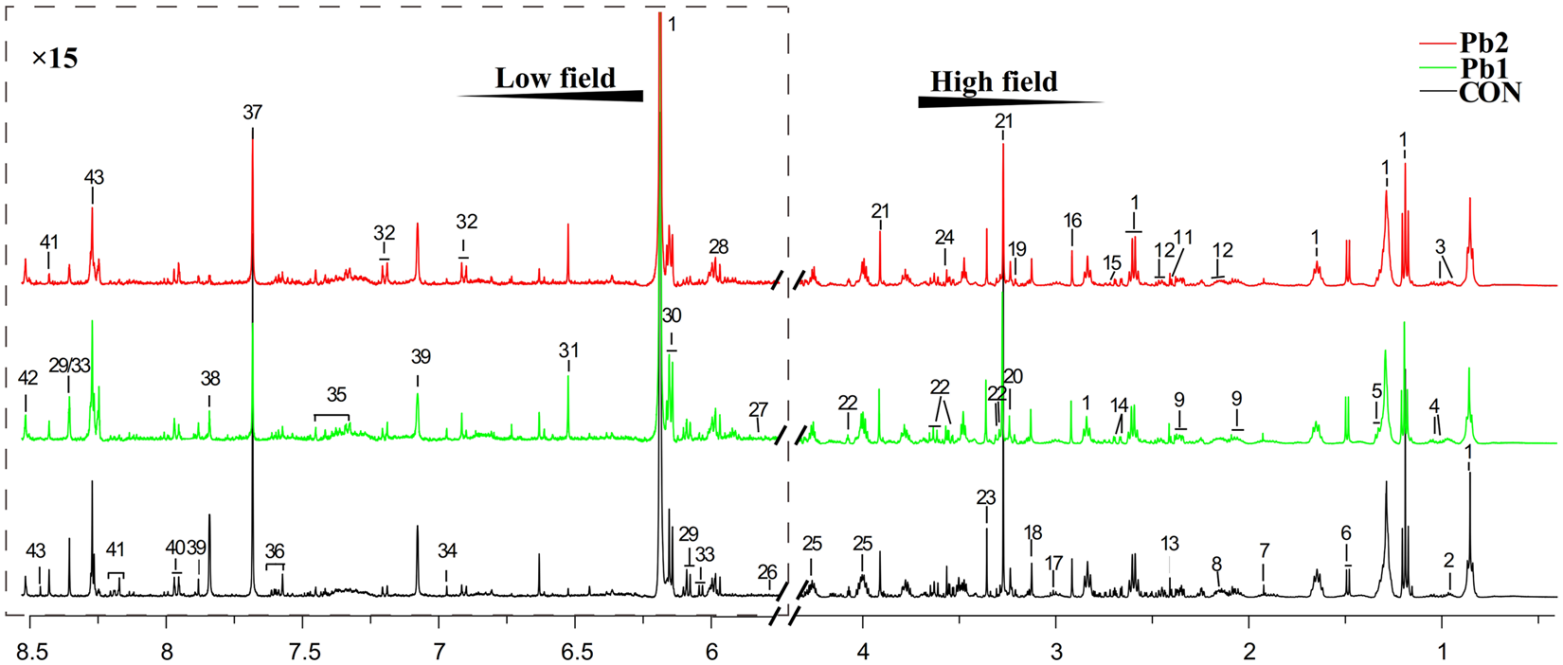
Representative 500 MHz ^1^H NMR spectra of earthworm polar extracts with metabolites labeled. CON, Pb1- and Pb2- treated groups in C soil are shown as example. “C” stands for cambosol. 1 2-hexyl-5-ethyl-3-furansulfonate (HEFS); 2 Leucine (Leu); 3 Isoleucine(Ile); 4 valine (Val); 5 Lactate(Lac); 6 Alanine(Ala) 7 Acetate (Ace); 8 Acetylcholine (Acec); 9 Glutamate (Glu); 10 Asparagine (Asp); 11 Pyruvate (Pyr); 12 Glutamine (Glm); 13 Succinate (Suc); 14 Malate (Mal); 15 Dimethylamine (DMA); 16 N,N-Dimethylglycine (DMG);17 Lysine (Lys); 18 Malonate (Malo); 19 Choline (Cho); 20 Glycerophosphocholine (GPC); 21 Betaine (Bet); 22 Myo-Inositol (MI); 23 Scyllo-Inositol (SI); 24 Glycine (Gly); 25 Lombricine (Lom); 26 Glucose (Gluc); 27 Maltose (Malt); 28 Uridine (Uri); 29 Inosine (Ino); 30 Adenosine Triphosphate (ATP); 31 Fumarate (Fum); 32 Tyrosine (Tyr); 33 Adenosine (Ade); 34 N,N-dimethylhistidine(DTH); 35 Phenylalanie (Phe); 36 Niacinamide (Nia); 37 t-Methylhistidine (MHis); 38 Dimethylxanthine (DMX); 39 Histidine (His); 40 UDP-glucose; 41 Nicotinamide Adenine Dinucleotide (NAD^+^)/Nicotinamide Adenine Dinucleotide Phosphate (NADP^+^); 42 Adenosine monophosphate (AMP); 43 Nicotinamide adenine dinucleotide (NADH)/Nicotinamide adenine dinucleotide phosphate (NADPH).

Typical ^1^H NMR spectra for the earthworm polar extracts from control, Pb1- and Pb2- treated groups in cambosol are presented in Fig. 2, with 43 metabolites assigned. Each earthworm from the heavy-metal- exposed group in ferrosol and cambosol had similar typical spectra, and we took Pb in cambosol as an example to show peaks and corresponding metabolites identified by NMR. Two-dimensional STOCSY analysis of ^1^H NMR spectra in earthworm polar extracts assisted in the identification of metabolites (Supplementary Fig. S1). Details for the labeled metabolites are shown in Supplementary Table S1. Metabolites were mainly related to amino acids, intermediates of the TCA cycle, neurotransmitter balance compounds, osmotic equilibrium and nucleic acid metabolism compounds.

### Multivariate statistical analysis of NMR spectra data

Unsupervised PCA and supervised OSC-PLS-DA were conducted on the binned ^1^H NMR metabolic data to obtain an overview of the variation among groups. Because it lacks class information, PCA is less able to capture variables that contribute to grouping than are supervised pattern recognition methods. Control and treated groups exhibited rough (Supplementary Fig. S2a,b and A) or no (Supplementary Fig. S2c and A) separation in the PCA score plot. In contrast, OSC-PLS-DA, which can distinguish important contributing variables from irrelevant noise, produced a score plot that clearly separated the control and exposed groups (Supplementary Fig. S2a–c and B). However, the OSC-PLS-DA score plots between C1 (Pb1 and Cd1) and C2 (Pb2 and Cd2) in each group showed partial overlap. The validity of the OSC-PLS-DA model among various exposed groups showed a satisfactory goodness of fit and good predictability (R^2^Y = 0.87, Q^2^Y = 0.56 for Pb in cambosol; R^2^Y = 0.79, Q^2^Y = 0.75; R^2^Y = 0.86, Q^2^Y = 0.78 for Pb and Cd in ferrosol, respectively; Supplementary Fig. S2a–c and D) and statistical significance (*p* = 0.0105 for Pb in cambosol and *p* = 0.0055, 0.003 for Pb and Cd in ferrosol, respectively; Supplementary Fig. S2a–c and C).

**Figure 3:**
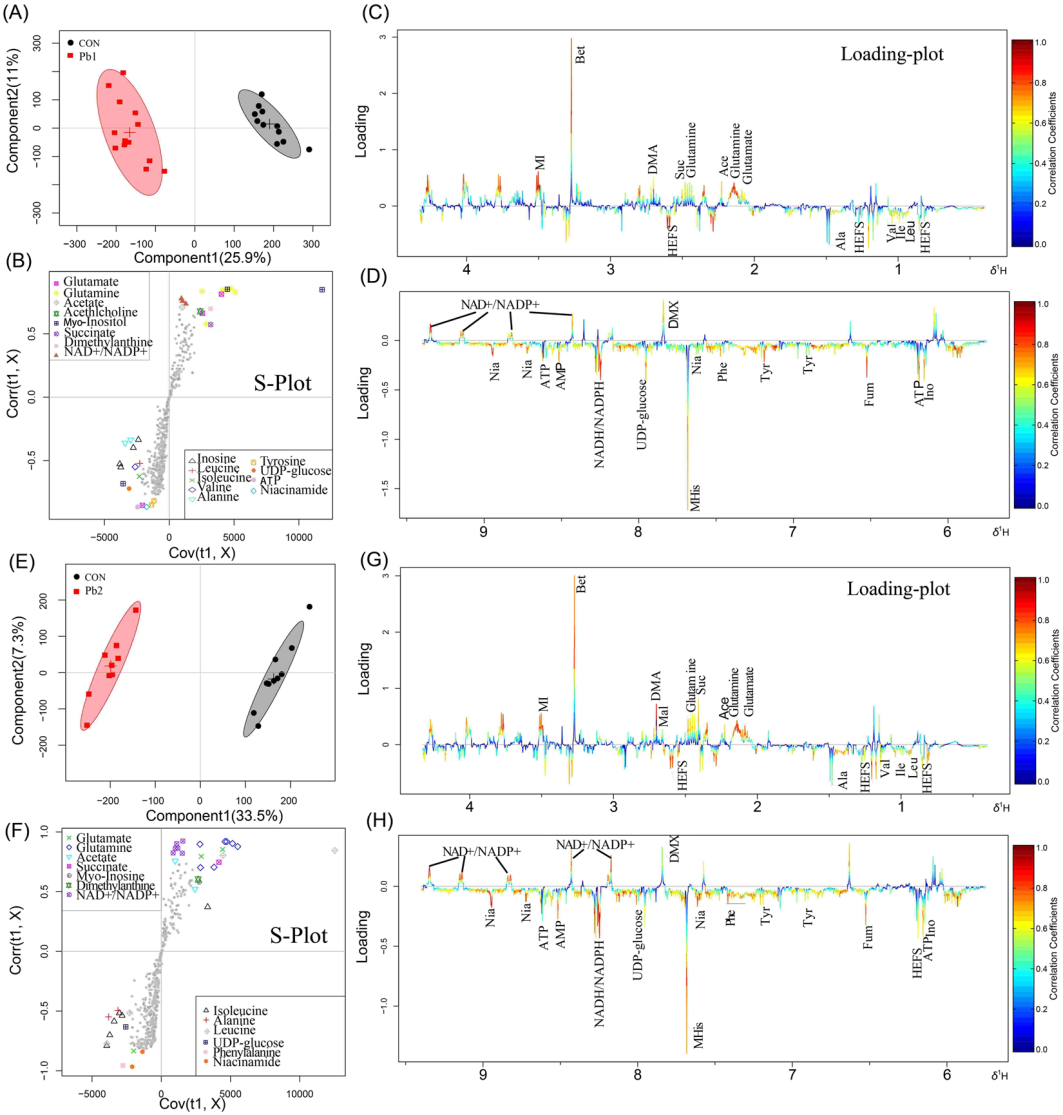
OSC-PLS-DA analysis of datasets from Pb1- treated and control (CON) group (**A**), Pb2- treated and CON group in cambosol (**E**). Score plot where one point represents one sample and one ellipse corresponds to a confidence interval of 95% and stand for a grouping (**C** and **F)**. S-plot where points represent different variables (metabolites). Loading plot (0.4–4.3 and 5.7–9.4 ppm) color is coded according to correlation coefficients from blue to red (**B**,**D**,**G**,**H**). The color bar corresponds to the weight of the corresponding variable in the discrimination of statistically significant (Red) or in significant (Blue). Positive and negative peaks indicate relatively decreased and increased metabolite levels in the Pb- treated groups.

**Figure 4:**
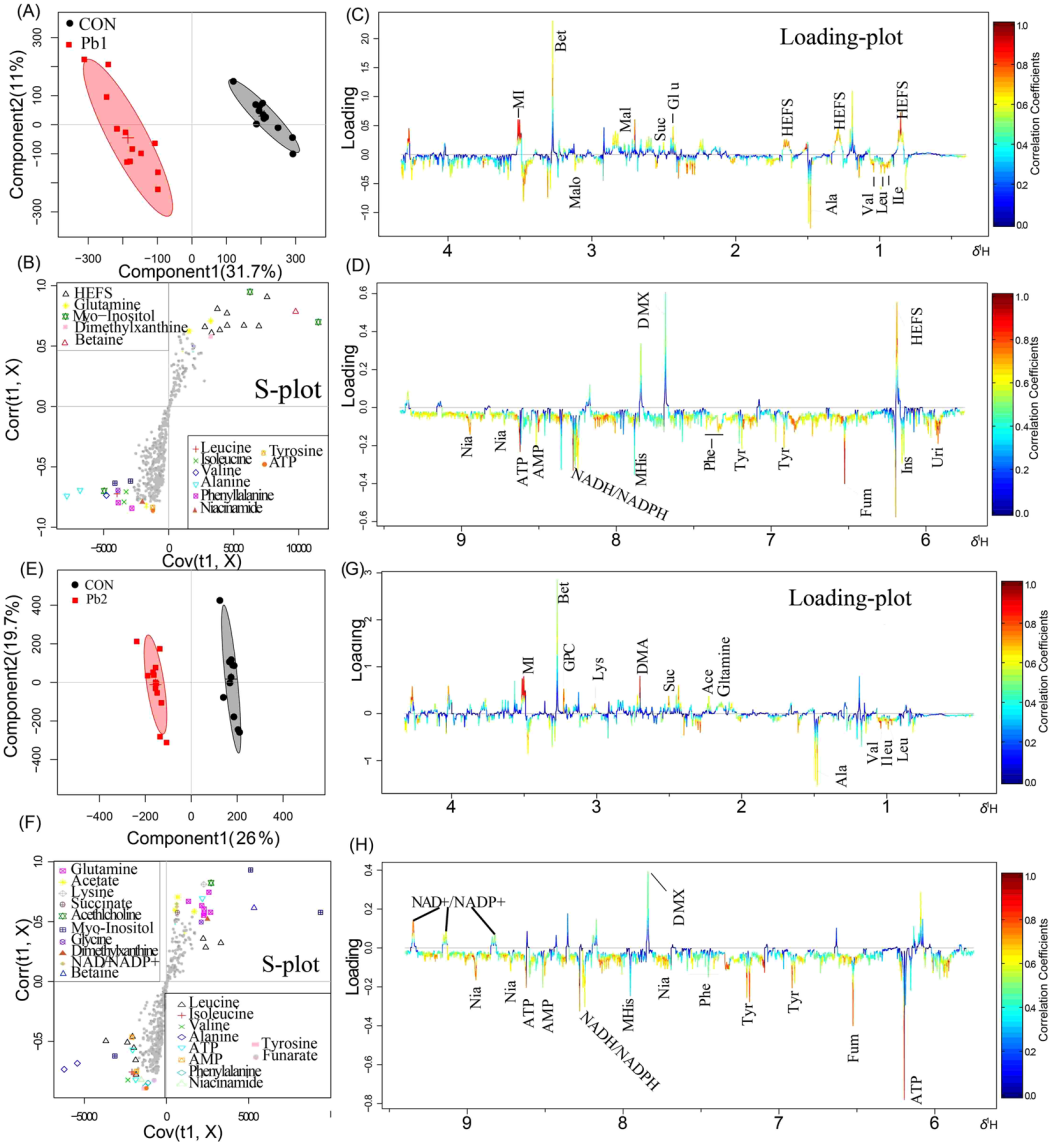
OSC-PLS-DA analysis of datasets from Pb1- treated and CON group (**A**), Pb2- treated and CON group in ferrosol (**E**). Score plot where one point represents one sample and one ellipse corresponds to a confidence interval of 95% and stands for a grouping (**C** and **F**), with an S-plot where points represent different variables (metabolites). Loading plot (0.4–4.3 and 5.7–9.4 ppm) color is coded according to correlation coefficients from blue to red (**B** and **D**,**G** and **H**). The color bar corresponds to the weight of the corresponding variable in the discrimination of statistically significant (Red) or in significant (Blue). Positive and negative peaks indicate relatively decreased and increased metabolite levels in the Pb treated groups.

**Figure 5:**
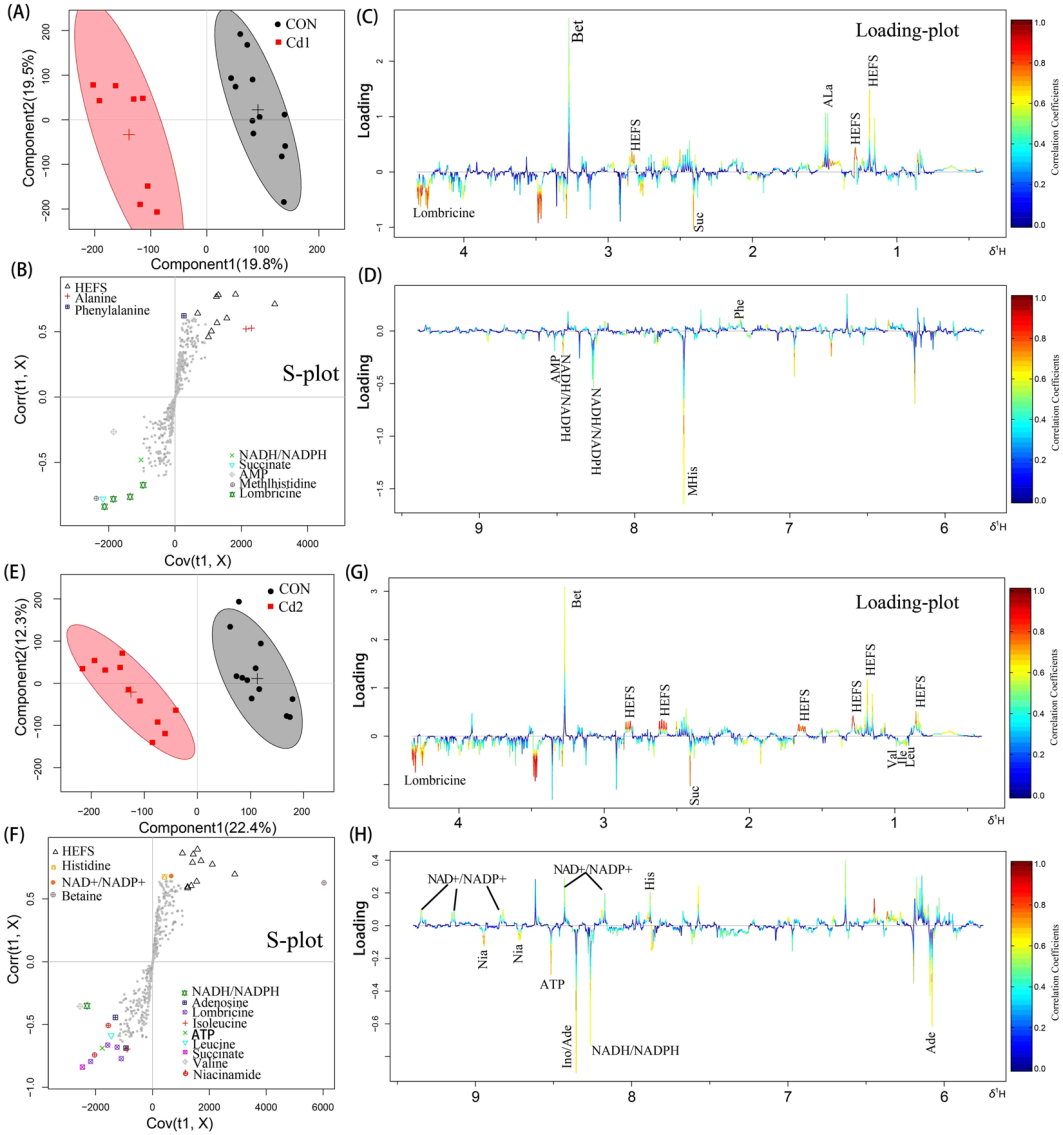
OSC-PLS-DA analysis of datasets from Cd1- treated and CON group (**A**), Cd2- treated and CON group in ferrosol (**E**). Score plot where one point represents one sample and one ellipse corresponds to a confidence interval of 95% stands for a grouping (**C** and **F**) and an S-plot where points represent different variables (metabolites). Loading plot (0.4–4.3 and 5.7–9.4 ppm) color is coded according to the correlation coefficients from blue to red (**B** and **D**, **G** and **H**). The color bar corresponds to the weight of the corresponding variable in the discrimination of statistically significant (Red) in significant (Blue). Positive and negative peaks indicate relatively decreased and increased metabolite levels in Pb- treated groups.

To determine which metabolites contributed to the model, loading plots of Pb-exposed groups in cambosol and ferrosol compared to the control group were conducted separately (Figs 3 and 4). The loading plots of Cd in ferrosol are presented in Fig. 5. All altered metabolites were mainly related to amino acids, osmolytes, neurotransmitters and TCA cycle compounds and nucleic acid metabolism, which are directly visualized by loading plots color coded according to the correlation coefficient and visualized in a covariance-based pseudo-spectrum^35^ (Figs 3–5C,D,G and H). Warm color indicates high significance for metabolites contributing to inter-class discrimination.

**Figure 6:**
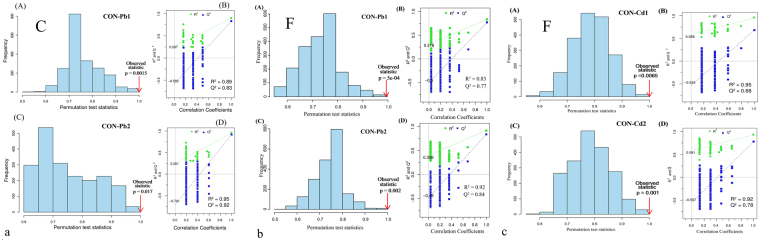
Histograms for permutation test scores of OSC-PLS-DA models based on 2000 permutations: red arrows indicate performance based on original labels, significant for a *p* value less than 0.05. ‘C’ and ‘F’ stand for cambosol and ferrosol.

Compared to the control group, earthworm metabolites in Pb-contaminated cambosol and ferrosol indicated that most amino acids such as leucine, isoleucine, valine, alanine, phenylalanine and tyrosine were significantly higher; osmolytes such as betaine and myo-inositol were significantly lower; compounds belonging to nucleic acid metabolism such as adenosine triphosphate (ATP), adenosine monophosphate (AMP), inosine and niacinamide were significantly higher; and TCA cycle intermediates, such as fumarate, were higher, and succinate was lower. Only a few metabolites had significantly detectable changes in the Cd-contaminated ferrosol. Metabolites with higher levels included leucine, isoleucine, valine, glutamate, dimethylamine, inosine and AMP, and those with lower levels included HEFS, histidine, betaine and nicotinamide adenine dinucleotide (NAD^+^)/nicotinamide adenine dinucleotide phosphate (NADP^+^). Significantly different metabolites in Pb-contaminated ferrosol are shown in Fig. 4. All metabolites shown to have different varying levels in this study are presented in Table 1. The OSC-PLS-DA models between each treated and control group showed good validity with R^2^Y = 0.89, Q^2^Y = 0.83, *p* = 0.0015 and R^2^Y = 0.92, Q^2^Y = 0.92, *p* = 0.017 in Pb1- and Pb2- contaminated cambosol; R^2^Y = 0.83, Q^2^Y = 0.77, *p* = 5e-04 and R^2^Y = 0.92, Q^2^Y = 0.84, *p* = 0.002 in Pb1- and Pb2- contaminated ferrosol; and R^2^Y = 0.95, Q^2^Y = 0.68, *p* = 0.0065 and R^2^Y = 0.92, Q^2^Y = 0.78, *p* = 0.001 in Cd1- and Cd2- contaminated ferrosol (Fig. 6).

**Table 1:** Fold changes of changing earthworm metabolites and *p* value in different Pb/Cd-contaminated soils compared to control group.

	

The fold changes of changing earthworm metabolites and *p* value in different Pb/Cd- contaminated soils compared with control group. Color is coded according to log_2_ (Fold) using color bar , and red and blue respectively stand for increase and decrease in metabolites in Pb- exposed group; *p*- values: *P < 0.05, **P < 0.01, ***P < 0.001. 1 2-hexyl-5-ethyl-3-furansulfonate (HEFS).

## Discussion

In this work, the metabolic responses of earthworms to different Pb/Cd-contaminated soils were characterized by a ^1^H NMR-based metabolomics approach along with a traditional metal accumulation approach. Cd in soils strongly accumulated in earthworms, whereas Pb in soils weakly concentrated in earthworms. Multivariate and univariate statistical analyses revealed that Pb/Cd-contaminated soils disturbed earthworm amino acid metabolism, osmotic equilibrium, nucleic acid metabolism neurotransmitter metabolism and TCA cycle. Furthermore, Pb and Cd-contaminated soils elicited distinct metabolic responses in earthworms.

### Pb/Cd accumulation and earthworm bioavailability

As earthworms ingest soil, Pb and Cd in the soil are taken up simultaneously by the earthworm gut. However, Pb and Cd may be treated in different ways in the special gut environment. After 14 days of exposure, the Cd concentrations in the earthworms were higher than those in the soil. In contrast, the Pb concentrations in the soil were higher than those in earthworms. The reported Cd and Pb-BAFs of earthworms in real contaminated pastures and roadsides are similar to this work^36–38^. Once absorbed, the meal may be metabolized and excreted, accumulated in other tissues, sequestered internally, or transported in the organism to the site of toxic action (STA)^39^. It is likely that the feeding Cd that is bound to soil in the earthworm digestive cavity can be rapidly transferred and accumulated in some earthworm tissues, as opposed to the slower transference and accumulation of Pb. However, as long as Cd and Pb bound to the soil were assimilated by the earthworms, their excretion might be slow or absent^37^. As a result, most Pb in the feeding soil of earthworms could be directly excluded from the earthworm intestinal tract, while Cd in soil could be assimilated by the earthworms. This finding may be attributed to the differences in the response mechanism of earthworms to lead and cadmium. Previous studies have shown that Cd can be bound by the earthworm metallothionein, which has abundant sulfhydryl groups that can function as bio-ligands, and accumulate in earthworms^40–43^, whereas Pb can be isolated by insoluble calcium phosphate combined with a phosphoryl group (another bio-ligand) in earthworms^44–46^. However, the related mechanisms need to be further studied.

The Pb concentrations and BAFs in Pb-contaminated ferrosol were greater than that in cambosol, likely due to the different physical and chemical properties of the two soils, resulting in differences in Pb bioavailability. Previous studies have also reported that the Pb concentration of the plant and CaCl_2_ extraction in ferrosol is higher than that in cambosol^47,48^, which indicates that Pb phytoavailability in ferrosol is obviously higher that than cambosol. Multiple linear regression analyses revealed that low pH and CEC in ferrosol could directly increase Pb phytoavailability^47^. However, the bioavailability of metals to animals is more complicated than phytoavailability owing to the complex gut environment. Reports indicated that gut conditions may decrease the number of metal binding sites (ligands) in the soil due to the chemical composition of gut liquid and the excretion of digestive enzymes^49^, which may alter metal bioavailability in the soil. Nonetheless, metabolomics can indirectly detect the animal availability of pollutants in the soil according to index of metabolic changes^50,51^. In this work, the earthworm metabolic profiles of Pb-contaminated ferrosol and cambosol were highly similar (Table 1), but the magnitude of the difference in most metabolites in ferrosol was obviously greater than that in cambosol. Cellular stress response is a defense reaction to environmental forces that is characteristic of all cells and may result in increased of the reactive oxygen species (ROS)^52^, deformation and damage of proteins, DNA or other essential macromolecules^53^ and changes of downstream metabolites. In this work, the higher Pb concentrations of earthworms in ferrosol than in cambosol may cause more serious stress and increase ROS, which leads to more intense metabolic changes. Therefore, we believe that Pb plant and animal availability shows similar trends in ferrosol and cambosol with greater availability in ferrosol based on the combined characterization of earthworm metabolomics (indirect) combined with a traditional Pb bioaccumulation approach (direct).

### Metabolic pathways of earthworms in different contaminated soils

Throughout the exposure period, we did not observe earthworms on the surface of the soil or any other signs of organism distress. The mortality in different groups did not exceed 10 percent at the end of the test period in line with the OECD reliable standard (90%) for earthworm exposure experiments^4^. However, the levels of various metabolites were higher or lower in Pb/Cd- contaminated soils than in the control group (Table 1).

Different pollutants may cause distinct changes in the same metabolite. In this work, the levels of acetylcholine, malate and dimethylamine decreased in Pb-contaminated soils, but they had no apparent differences in Cd-contaminated soil. In addition, the level of fumarate presented the opposite changes in Pb/Cd-contaminated soils. Furthermore, after earthworms are exposed to artificial soil polluted with endosulfan sulfate, the glutamine levels are higher in tissue extracts than in the control group^54^. The level of glutamine decreased in Pb-contaminated ferrosol and cambosol and was not obviously different in Cd-contaminated ferrosol. However, metabolites may present the same changes in earthworms exposed to different pollutants. For example, when earthworms are exposed to a commercially available clay loam soil with pyrene pollution, the levels of alanine, leucine, valine, isoleucine and tyrosine are higher than those in controls^19^, consistent with the Pb- contaminated ferrosol and cambosol in this study. Therefore, changes to individual metabolites cannot give detailed information on the metabolic mechanism of earthworms. A system analysis approach to metabolites must be developed to better understand the effects of pollutants on metabolic processes.

**Figure 7:**
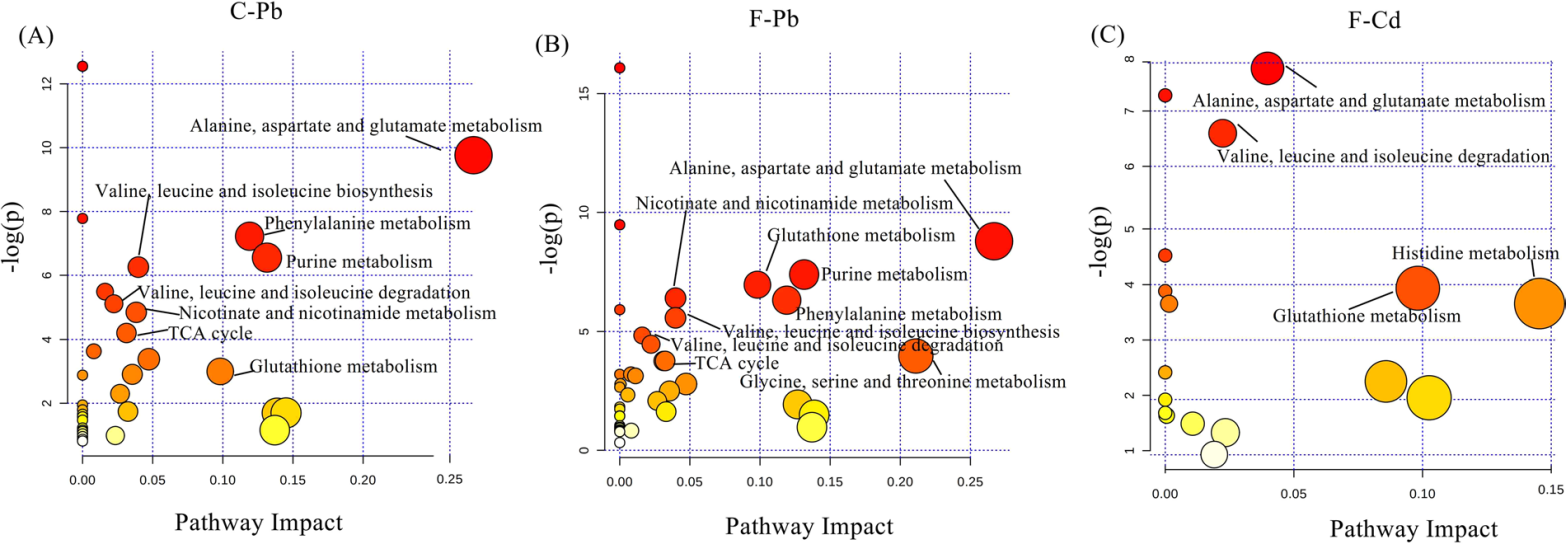
Overview of affected metabolic pathways of Pb- and Cd- contaminated soils to earthworms compared with CON as visualized by bubble plots. Each bubble stands for one metabolic pathway. The area is proportional to the impact of each pathway, with color denoting the significance from highest (red) to lowest (white). Impact is pathway impact value calculated from pathway topology analysis. A-C, respectively, presents the disturbing metabolism of Pb- contaminated cambosol and ferrosol and Cd- contaminated ferrosol.

There is not yet a “metabolite ontology” database that can systematically annotate single metabolites into others^55^. To some extent, online MetPA analysis can help characterize the most relevant metabolic pathways involved in the toxicity of Pb- and Cd- contaminated soils to earthworms (Fig. 7). Related parameters for MetPA are shown in the Supporting Information as Excel profiles (Supplementary Tables S2–S4). Intriguingly, the alanine-aspartate-glutamate metabolism of each treated group was influenced and can be considered potential universal biomarkers for characterizing the toxicity of Pb/Cd-contaminated soils at the molecular level. Most pathways influenced in Pb-contaminated ferrosol and cambosol were alike and concerned alanine-aspartate-glutamate, purine, glutathione, valine-leucine-isoleucine biosynthesis and degradation, TCA cycle and nicotinate-nicotinamide metabolism, except for glycine-serine-threonine metabolism. Although the soil types are different, the toxic mechanisms of Pb-contaminated soils to *Eisenia fetida* earthworms are similar and have the same adverse outcome pathway (AOP). Most altered metabolites in Pb1- and Pb2- exposure groups seldom presented opposite changes compared to the control group, which not only mutually validates the reliability of the experimental results but confirmed the same AOP of Pb1 and Pb2 to earthworms. However, effective metabolic pathways for earthworms in Cd-contaminated ferrosol concerned alanine-aspartate-glutamate metabolism, valine-leucine-isoleucine degradation, histidine metabolism and glutathione metabolism, which were evidently less than and different from Pb-contaminated ferrosol. Although the soil type was the same, different heavy metals (Cd and Pb) can affect different metabolic pathways in the earthworms.

Correlation analysis of metabolite fold changes also supported the results described above in terms of the metabolic responses of earthworms to Pb/Cd-contaminated soils (Supplementary Fig. S3). The correlations between the fold changes of metabolites in Pb-contaminated ferrosol and cambosol were significant and had a high determination coefficient (R^2^ = 0.76, *p* < 0.0001; Supplementary Fig. S3A). The metabolite correlations in both Pb and Cd-contaminated ferrosol indicated a very low determination coefficient, but the *p* value was 0.02 exhibiting statistical significance (Supplementary Fig. S3B). Further, we conjectured that metabolic changes of earthworms in heavy metal-contaminated soils may be influenced by not only the heavy metals in the soil but also the physical and chemical properties of the soil. For example, previous studies have indicated that different organic matter content will influence the toxicity of pollutants to earthworms^56^. Notably, the partial overlaps of OSC-PLS-DA scores between dose 1 and dose 2 showed that a metabolic response range of the earthworms *Eisenia fetida* may be similar with slight differences. It has been reported that there is a possible concentration threshold of pollutants when the organism presented a higher toxicity^16,39^. To explore possible dose-effects to heavy-metal-contaminated soils on earthworm metabolism with the ^1^H NMR metabolomics approach, dose-response experiments must be performed in future studies.

Overall, these findings can be helpful not only to explore the relative metabolic mechanisms of *Eisenia fetida* earthworm to Pb/Cd-contaminated soils but also to provide a basis for further studies in terms of exploring factors that influence the metabolic pathways of earthworms in low heavy-metal-contaminated soils. However, it should be noted that we cannot systematically explain the toxic mechanism of Pb/Cd-contaminated soils to earthworms due to the absence of biochemical indexes (e.g., total glutathione and catalase) and other omics data (e.g., genome and transcriptome). These research efforts are ongoing and will be reported in the future. The ^1^H NMR based metabolomics approach can be used to investigate the environmental toxicity of low heavy metal contaminated soils, which is still in early stages in soil science, but has a promising role there due to its advantages. For example, its low cost enables gain high-throughput testing of metabolites with good reproducibility and the possible toxic mechanism at the molecular level may be explained. Above all, this method can also be used as a supplement to traditional toxic test approaches.

## Materials and Methods

### Soil preparation

In the present study, cambosol (C) derived from alluvial deposits was collected from the Baodi district of Tianjin, China, and ferrosol (F) derived from quaternary red clay was gathered from the Ecological Experimental Station of Red Soil, Chinese Academy of Sciences, Yingtan City, Jiangxi Province, China. The basic physical and chemical parameters of the two soils are given in Supplementary Table S5 (control plus two metal addition concentrations to soils). In May 2010, Pb(NO_3_)_2_ and 3CdSO_4_.8H_2_O solutions at three different concentrations were added to ferrosol and cambosol, depending on the pH (<6.5 and 6.5~7.5) of the soil and the Pb/Cd limit of the second-grade standard value of the National Soil Environmental Quality Standard of China (NSEQSC, GB 15618–1995; Pb: control [CON], half-and one-fold; Cd: CON, one- and two-fold). Only one kind of metallic salt solution rather than a mixture of heavy metals was added to each soil type (Supplementary Table S6).

**Figure 8:**
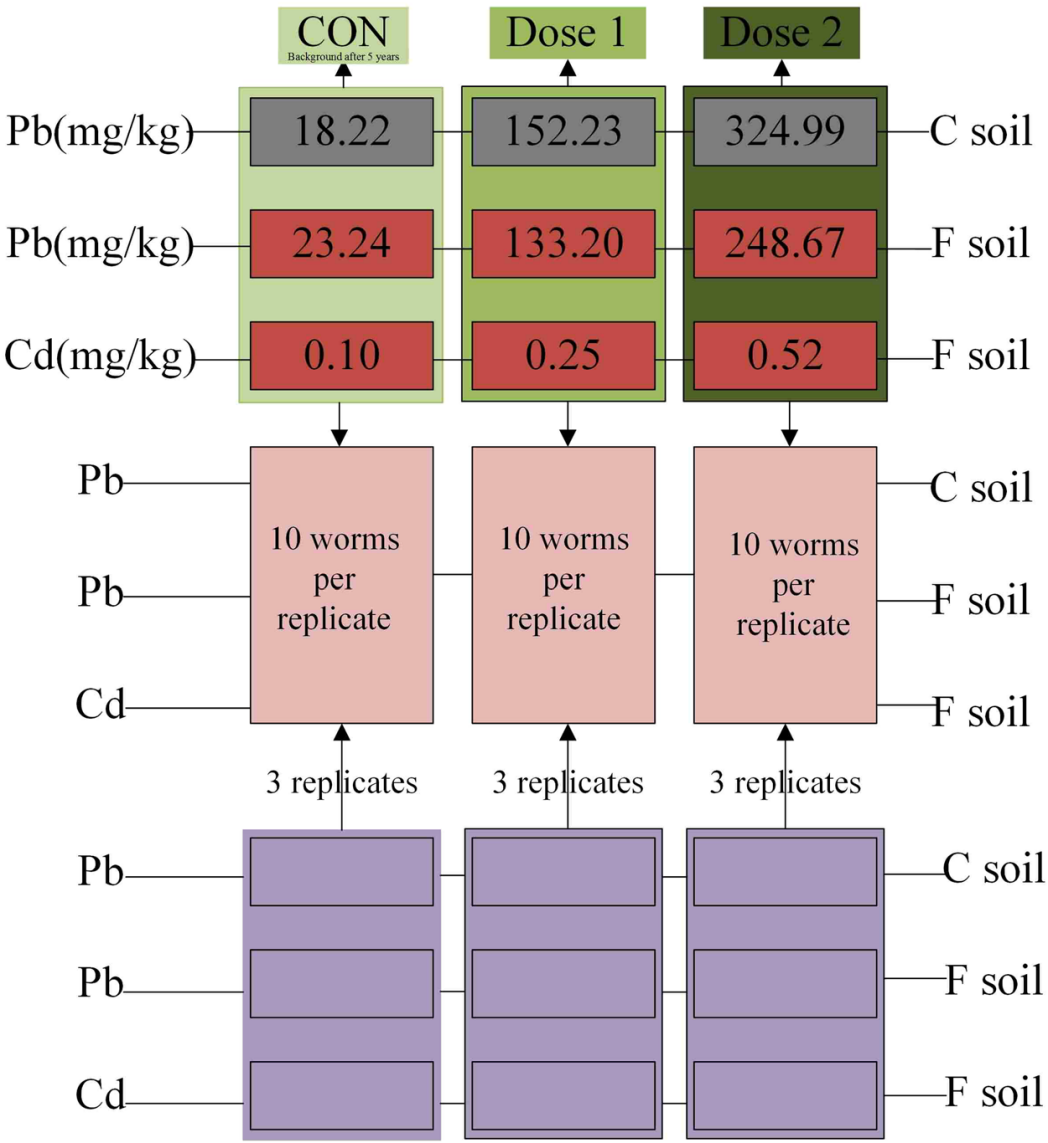
Schematic representation of experimental design for heavy metal exposure in this study. The figure shows three doses for each heavy metal exposure in ferrosol (F) and cambosol (C), together with the number of true biological replicates, each container (technical replicate) contains 10 earthworms. Each treated group included three replicates.

Five-year-aged Pb/Cd-contaminated soils, which were equal to historically contaminated soils, were milled using a wooden stick and sieved to 2 mm. In October 2015, the concentrations of the stored ferrosol and cambosol without and with Pb treatment were 23.24 ± 0.29, 133.20 ± 0.18, 248.67 ± 10.75 mg/kg (F: CON, Pb1and Pb2) and 18.22 ± 0.16, 152.23 ± 10.56 and 324.99 ± 15.22 (C: CON, Pb1 and Pb2) mg/kg, respectively; the concentrations of archived ferrosol without and with Cd treatment were 0.100 ± 0.002,0.252 ± 0.011 and 0.524 ± 0.024 mg/kg (F: CON, Cd1 and Cd2; Fig. 8). A total of 500 g of soil was poured into a 1 L glass containers that had been washed with dilute nitric acid and deionized water to remove binding metals from the glass wall; the soil was evenly mixed with deionized water. The soil humidity was kept at approximately 35% of dry soil weight by spraying an appropriate amount of deionized water on the soil surface each day according to the whole weight of the soil, water, earthworms and beaker.

### Earthworm exposure

Earthworms were purchased from a worm factory in Jiangsu Province, China. To reduce the effects of diet and other environmental factors, earthworms were raised in a constant temperature incubator at 20 °C for one month and fed cattle mature from an organic farm to acclimate to the laboratory environment before exposure experiments.

Mature earthworms with a well-developed clitellum were selected and depurated on wet filter paper for 48 h and the filter paper was replaced every two hours. Then, without adding food, ten earthworms were placed into each exposure group with three replicates (Fig. 8). The culture containers were covered with double gauze for two weeks to prevent the exposed medium from drying. The temperature and relative humidity were maintained at 20 ± 1 °C and 80% with a light intensity of 600 Lux and controlled by an artificial intelligence incubator. Exposure conditions were based on the OECD guidelines, apart from the exposed soils^4^.

### Analysis of metal concentrations in the whole earthworm body

Three earthworms on the 1st, 7th and 14th days of exposure were chosen and analyzed. Thereafter, each earthworm was kept on wet filter paper for 96 h to void their gut contents due to slow depuration kinetics for Cd and Pb^57^ to reduce the bias from heavy metal in residual soil in the gut. The process is illustrated in Supplementary Fig. S4. The remaining earthworms in each group were collected on the 14^th^ day and their guts were voided for NMR analysis. Each fresh earthworm was weighed and the survival rate was calculated by means of the numbers of deaths during the entire exposure period by dividing into the initial total numbers in three replicates. For the metal analysis, the three earthworms were rinsed, flash-frozen, lyophilized (48 h), weighed and ground. The powder from each earthworm (n = 27) was digested independently using HNO_3_ and H_2_O_2_ (GB 5009.12-2010). The digests were diluted appropriately with ultrapure water and analyzed using inductively coupled-plasma mass spectrometry (ICP-MS). The recoveries were 97.8–103.7% for the reference materials (GBW10049, Green Chinese onion, National Quality and Technology Supervision Agency of China), and the coefficient of variation (CV) was below 5%. Lead and Cd bioaccumulation factors (BAFs) of earthworms were calculated as the metal concentration of earthworms (dry weight) divided by the metal concentration of the soil.

### Solvent extractions of earthworm tissues

Ten to twelve earthworms per group were rinsed with distilled water to remove the residual soils on the skin surface and were depurated for 48 h on filter paper in 500 mL beakers. Earthworms were snap-frozen in liquid nitrogen, lyophilized and stored at −80 °C in a freezer before extraction^58^. Lyophilized earthworms were homogenized in 5 mL centrifuge tubes in an ice bath using a portable tissue homogenizer with a 6 mm stainless steel spatula (portable tissue homogenizer, S10, Ningbo, China)^20^. The homogenized earthworms were extracted according to a two-step extraction protocol^59^. First, ice-cold solvent (methanol: water = 4:0.85, v/v) was added to the tissue and vortexed for approximately 15 s. Second, ice-cold chloroform (4 mL/g dry earthworm) and water (2 mL/g dry earthworm) were added and vortexed for 60 s. The mixtures were partitioned into polar and nonpolar layers after standing in ice for 10 min and centrifuged at 12,000 rpm for 10 min at 4 °C. The upper polar solvent phase was transferred to a new 2 mL centrifuge tube, the organic phase was evaporated under nitrogen, and the remaining aqueous phase was frozen and lyophilized to dryness in a vacuum freeze-drying concentrator. The lyophilized samples were dissolved in 750 μL of 99.9% D_2_O phosphate buffer (0.2 M, pH 7.4) containing 0.1% (w/v) sodium azide (99.5% purity; Sigma-Aldrich) as a preservative. Then, 0.05% (w/v) sodium 3-(trimethylsilyl) propionate-2,2,3,3-d_4_ (TSP, Sigma Aldrich) was added to the buffer solution as an internal standard (^1^H, 0.00 ppm). The samples were vortexed and centrifuged at 12000 rpm for 10 min. Finally, the supernatants were transferred to 5 mm NMR tubes for ^1^H NMR analysis^60–62^.

### ^1^H NMR spectroscopy

All ^1^H NMR spectra were acquired at 298 K on a Bruker Avance 500 MHz flow-injection spectrometer (Bruker GmbH, Karlsruhe, Germany) using a 5 mm PABBO BB-inverse gradient probe. A modified transverse relaxation-edited Carr-Purcell-Meibom-Gill (CPMG) sequence (RD-90(τ-180-τ)n-ACQ) with a total spin-echo delay (2 nτ) of 10 ms was used to suppress the signals of possible residual macromolecules and partial water peaks. Typically, 128 scans were collected into 32 K data points using a spectral width of 10000 Hz. The spectra were Fourier transformed after multiplying the FIDs by an exponential weighting function corresponding to a line-broadening of 0.5 HZ.

### Data processing

All NMR spectra were manually adjusted for phase and baseline, aligned to TSP (δ = 0.0 ppm) using Topspin 3.0 software (Bruker GmbH, Karlsruhe, Germany) and exported as ASCII files using MestReNova (Version 8.0.1, Mestrelab Research SL, Santiago de Compostela, Spain). The files were imported into the open-source software “R” for further phase and baseline correction, and peak alignment. After removing the signals for water and its affected neighboring regions (4.70–5.25 ppm), the NMR data were binned using an adaptive binning approach^63^ from 0.2 to 8.8 ppm with an average bin width of 0.015 ppm. All binned spectra were mean-centered, and the integral values of each spectrum were probability quotient normalized before multivariate statistical analysis to simplify the interpretation of coefficients in all models^64^.

Most metabolites were identified using the software Chenomx NMR Suite 7.7 (Chenomx Inc., Edmonton, Canada) by querying the publicly accessible metabolomics databases such as the Human Metabolome Database (HMDB, http://www.hmdb.ca). However, some overlapping metabolites in the NMR spectra were distinguished by representative references^24,58,62,65,66^. The indistinguishable overlapping peaks of different metabolites hampered the accurate assignments of NMR signals and peak integrations; thus, the assignment and integration of peaks were assisted by two-dimensional statistical total correlation spectroscopy (STOCSY), which was used to identify correlations between spectral resonances of interest to assist metabolite assignment^67^. Understandably, different resonances belonging to the same molecules were highly correlated, which helped assign metabolites. Metabolic pathway analysis (MetPA) was conducted using Metaboanalyst (http://www.metaboanalyst.ca) to reveal the most relevant metabolic pathways that were disturbed^68^. Only significantly different metabolites were selected for MetPA analysis and over-representation analysis based on hypergeometric test and pathway topology analysis based on relative betweenness centrality algorithms were selected for calculations. Raw *p* is the original *p* value calculated from the enrichment analysis.

### Data statistical analysis

Multivariate statistical analyses, including unsupervised principal component analysis (PCA) and supervised orthogonal signal correction-partial least squares-discriminant analysis (OSC-PLS-DA), were executed using in-house-developed scripts in “R” (http://cran.r-project.org/). OSC-PLS-DA was conducted to separate different groups. Orthogonal signal correction (OSC) was used to remove uninteresting variation, such as systematic variation, from the NMR spectra prior to PLS-DA. The OSC-PLS-DA model was validated and assessed by 2000-times permutation testing and repeated two-fold cross-validation (2CV). The observed *p*-values (*p* < 0.05) via permutation testing confirmed the significance of the OSC-PLS-DA model at a 95% confidence level. The performance measures were plotted on a histogram for visual assessment. Cross-validation was evaluated by the parameters R^2^Y and Q^2^Y, which were used to avoid overfitting and reflect the predictability of the model. Generally, higher Q^2^Y values (>0.5) indicated significant differences among groups. Color-coded loading plots were used to identify significantly changing metabolites; metabolite signals represented by warm colors contribute more to class differentiation than those with cold colors. The fold change values of metabolites and associated *p*-values corrected by the Benjamini & Hochberg adjusted method were calculated for multiple comparisons and visualized in the colored table^69^. Fold change values were calculated by means of dosed group/control group and color-coded after logarithmic (log) transformation. The red color indicates increasing metabolites and the blue color indicates decreasing metabolites in the earthworm. Student’s *t* test was conducted to show significant metabolites that were higher or lower between the groups, and *p* < 0.05 was considered statistically significant.

## Electronic supplementary material

Supporting information

Table S2

Table S3

Table S4
