# Supplementary material for: Metabolic Responses of Eisenia Fetida to Individual Pb and Cd Contamination in Two Types of Soils
Source: Sci Rep. 2017 Oct 12;7:13110. doi: 10.1038/s41598-017-13503-z (PMC5638831; doi:10.1038/s41598-017-13503-z)
Supplement: Supplementary file 1 — Supporting information [file 41598_2017_13503_MOESM1_ESM.pdf]

# Supporting information for

# "Metabolic Responses of *Eisenia Fetida* to Individual Pb and Cd Contamination in Two Types of Soils"

**Ronggui Tang[1, 4], Changfeng Ding[1], Yibing Ma[2], Junsong Wang[3, *], Taolin Zhang[1], XingxiangWang[1*]**

1. Key Laboratory of Soil Environment and Pollution Remediation, Institute of Soil Science, Chinese Academy of Sciences, Nanjing, 210008, People's Republic of China
2. Institute of Agricultural Resources and Regional Planning, Chinese Academy of Agricultural Sciences, Beijing, 100081, People's Republic of China
3. Center for Molecular Metabolism, School of Environmental and Biological Engineering, Nanjing University of Science and Technology, Nanjing, 210014, People's Republic of China
4. University of the Chinese Academy of Sciences, Beijing, 100049, People's Republic of China

**Corresponding Author**

* Prof. Xingxiang Wang and Junsong Wang. E-mail address: xxwang@issas.ac.cn (Xingxiang Wang), junsong@gmail.com (Junsong Wang), Tel.: +86-02586881200 (Xingxiang Wang)

Table S1. Metabolites identified from the polar tissue extracts of earthworms.

| NO. | Metabolites | Assignments | Chemical shift (ppm) | |
|---|---|---|---|---|
| | | | H | C |
| 1 | HEFS | | 0.85(t), 1.19(t),1.28(m), 1.30(m),1.64(q),2.60(m),2.83(t),6.19(s) | |
| 2 | Isoleucine | 9’$CH_3$ | 0.99(d) | 17.37 |
| 3 | Leucine | 8’$CH_3$, 9’ $CH_3$ | 0.95(t) | 24.80 |
| 4 | Valine | 7’ $CH_3$ | 1.03(d) | 20.75 |
| 5 | Lactate | 3’$CH_3$ | 1.32(d) | 22.90 |
| 6 | Alanine | 6’ $CH_3$ | 1.48(d) | 19.03 |
| 7 | Acetate | 4’ $CH_3$ | 1.91(s) | 24.64 |
| 8 | Acetylcholine | 8’$CH_3$ | 2.15(s) | 22.95 |
| 9 | Glutamate | 6’$CH_2$,7’$CH_2$ | 2.04(m),2.34(m) | |
| 10 | Asparagine | 6’$CH_2$ | 2.94(m) | 37.43 |
| 11 | Pyruvate | 6’$CH_3$ | 2.36(s) | 29.23 |
| 12 | Glutamine | 6’$CH_2$,7’$CH_2$ | 2.13(m),2.45(m) | 29.29,33.93 |
| 13 | Succinate | 4’$CH_2$,5’$CH_2$ | 2.39(s) | 36.83 |
| 14 | Malate | 5’$CH_2$ | 2.66(dd) | 45.46 |
| 15 | Dimethylamine | 2’$CH_3$,3’$CH_3$ | 2.72(s) | 37.30 |
| 16 | Dimethylglycine | 6’$CH_3$,7’$CH_3$ | 2.91(s) | 46.35 |
| 17 | Lysine | 8’$CH_2$, 9’ $CH_2$ | 1.71(m), 3.02(t) | 29.15,42.12 |
| 18 | Malonate | 4’$CH_2$ | 3.12(s) | 50.23 |
| 19 | Choline | 5’$CH_3$,6’$CH_3$,7’$CH_3$ | 3.19(s) | 56.70 |
| 20 | Glycerophosphocholine | 5’$CH_3$,6’$CH_3$,13’$CH_3$ | 3.22(s) | 56.64 |
| 21 | Betaine | 3’$CH_2$, 5’$CH_3$, 7’$CH_3$, 8’$CH_3$ | 3.25(s),3.89 (s) | 55.86,68.64 |
| 22 | Myo-Inositol | 2’CH, 6’CH, 3’CH, 5’CH, 1’CH | 3.27(m),3.52(dd),3.61(t),4.05(t) | 77.16,73.96,75.13,74.93 |
| 23 | Scyllo-Inositol | | 3.35(s) | |

| | | | | |
|---|---|---|---|---|
| 24 | Glycine | 4’$CH_2$ | 3.54(s) | 44.30 |
| 25 | Lombricine | | 3.48(t), 4.26(dd) | |
| 26 | Glucose | 2’CH | 5.22(d) | 94.94 |
| 27 | Maltose | 2’CH | 5.40(d) | 102.28 |
| 28 | Uridine | 2’CH,10’CH | 5.88(d),5.90(d) | 105.08,92.09 |
| 29 | Inosine | 2’CH | 6.06(d) | 90.96 |
| 30 | ATP | 2’CH,12’CH,7’CH | 6.13(d),8.24(s),8.53(s) | 89.28,155.37,142.50 |
| 31 | Fumarate | 4’CH,5’CH | 6.51(s) | 138.00 |
| 32 | Tyrosine | 2’CH, 6’CH, 3’CH, 5’CH | 6.88(m),7.17(m) | 118.89,133.49 |
| 33 | Adenosine | 2’CH | 6.02(d) | 91.06 |
| 34 | N,N-dimethylhistidine | | 7.08(s) | |
| 35 | Phenylalanine | (1-6)’CH | 7.32(d),7.36(m),7.42(m) | 132.12,130.43,131.81 |
| 36 | Niacinamide | 5’CH, 6’CH, 2’CH | 7.58(dd),8.70(dd),8.92(s) | 126.84,154.53,150.36 |
| 37 | τ-Methylhistidine | 2’CH | 7.67(s) | 141.12 |
| 38 | Dimethylxanthine | 7’CH | 7.87(s) | |
| 39 | Histidine | 5’CH, 2’CH | 7.09(d),7.90(d) | 119.99,138.37 |
| 40 | UDP-glucose | 32’CH | 7.95(d) | 144.19 |
| 41 | NAD+/ NADP+ | | 8.21(m),8.41(s),8.84(d),9.15(d),9.33(s) | 131.14,142.42,148.39,145.07,142.51 |
| 42 | AMP | 7’CH | 8.58(s) | 142.61 |
| 43 | NADH/NADPH | | 8.46(s) | |

Multiplicity: (s) singlet, (d) doublet, (t) triplet, (q) quartets, (m) multiplets, (dd) doublet of doublets.

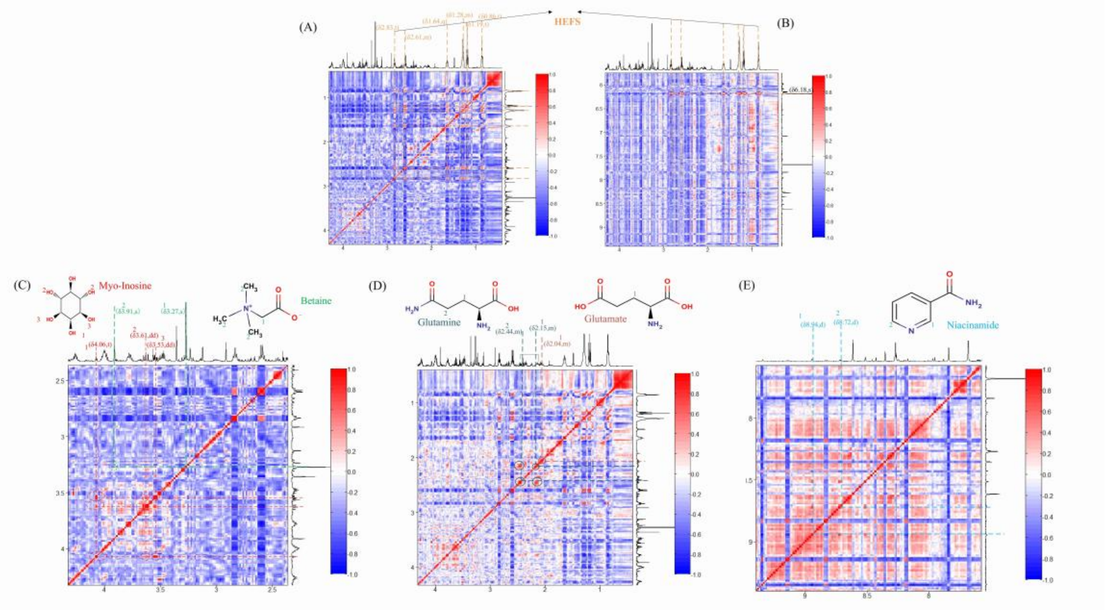


Figure S1 Example two-dimensional STOCSY analysis of $^1$H NMR spectra of earthworm polar extracts to aid the identification of metabolites. (A) and (B) 2D STOCSY subplots from 0.4-4.3~ 0.4-4.3 and 0.4-4.3~5.75-9.4 ppm for the assignments of HEFS; (C) 2D STOCSY subplots from 2.36 to 4.23 ppm for the assignments of myo-inosine and betaine. (D) 2D STOCSY subplots from 0.4 to 4.3 ppm for the assignments of Glutamine and glutamate. (E) 2D STOCSY subplots from 7.57 to 9.4 ppm for the assignments of niacinamide.

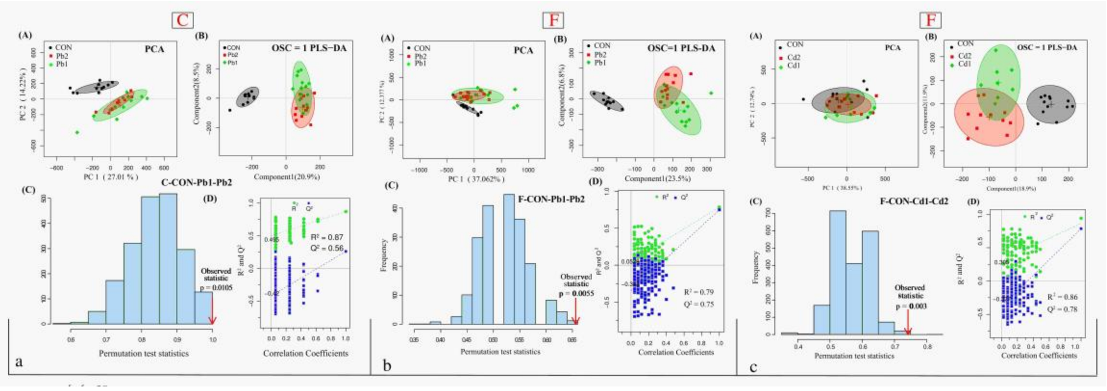


Figure S2 PCA and OSC-PLS-DA analysis of datasets from earthworm extracts among high-dosed, low-dosed and control groups of C and F soils (Figure S3 a, b, c (A) and (B)). Histograms for permutation test scores of OSC-PLS-DA models on the basis of 2000 permutations: the red arrows indicate the performance based on the original labels, significant for a P-value less than 0.05 (Figure S3 a, b, c (C)). OSC-PLS-DA scatter plot of the statistical validations obtained by 2000 times permutation tests, with $R^2$ and $Q^2$ values in the vertical axis, the correlation coefficients (between the permuted and true class) in the horizontal axis, and the ordinary least squares (OLS) line

for the regression of $R^2$ and $Q^2$ on the correlation coefficients (Figure S3 a, b, c (D)).

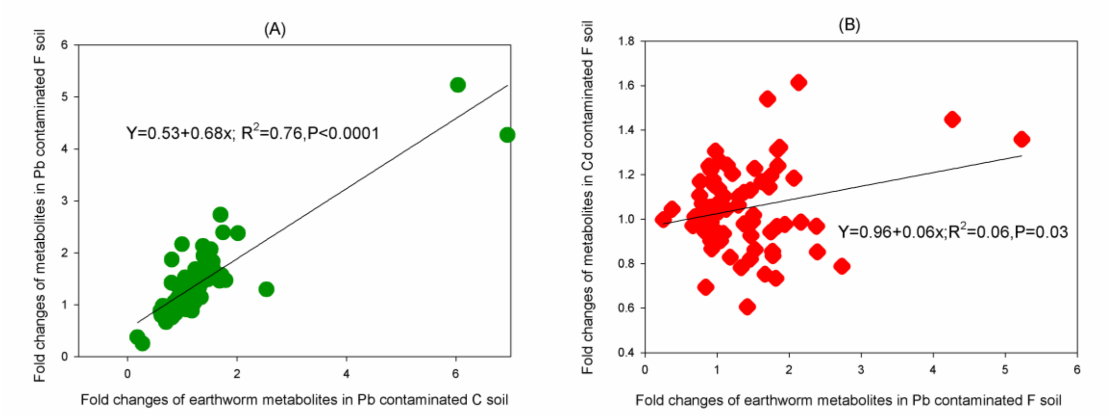


Figure S3. The correlation analysis of metabolites fold changes in different heavy metals contaminated soils. A and B respectively showed the correlation of metabolite fold changes in Pb contaminated F and C soils, Pb and Cd contaminated F soil.

Table S5. Basic physical and chemical properties of the exposed soil

| Soil types | pH | OC ($g.kg^{-1}$) | CEC ($cmol.kg^{-1}$) | Particles (SI) | | | Background value (mg/kg) | |
|---|---|---|---|---|---|---|---|---|
| | | | | Sand | Silt | Clay | Pb | Cd |
| Ferrosol (F) | 4.84 | 5.43 | 9.31 | 32.3 | 21.9 | 45.8 | 18.2 | 0.12 |
| Cambisol (C) | 6.93 | 9.90 | 24.1 | 19.4 | 44.1 | 36.5 | 23.2 | |

Table S6. Added metallic salt solution at each independently treated group

| Soil types | Added Pb(mg/kg) | | | | Added Cd(mg/kg) | | |
|---|---|---|---|---|---|---|---|
| | CON | Pb1 | Pb2 | | CON | Cd1 | Cd2 |
| Ferrosol (F) | 0 | 125 | 250 | F | 0 | 0.3 | 0.6 |
| Cambisol (C) | 0 | 150 | 300 | C | | — | |

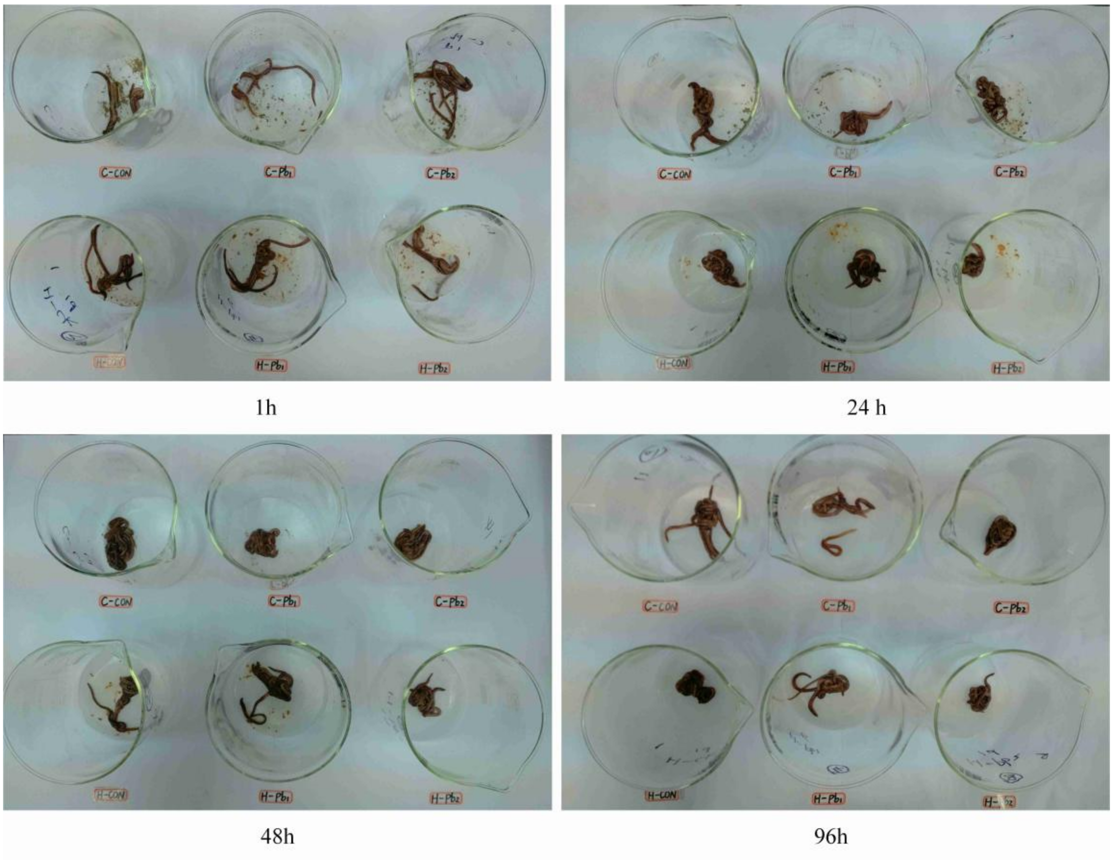


Figure S4. The clear process of earthworm gut. In fact, we observed once every two hours and replaced the soiled filter paper until there is no earthworm emissions on the filter paper.
